# Over-expression of microRNA-1 causes arrhythmia by disturbing intracellular trafficking system

**DOI:** 10.1038/srep46259

**Published:** 2017-04-11

**Authors:** Xiaomin Su, Haihai Liang, He Wang, Guizhi Chen, Hua Jiang, Qiuxia Wu, Tianyi Liu, Qiushuang Liu, Tong Yu, Yunyan Gu, Baofeng Yang, Hongli Shan

**Affiliations:** 1 Department of Pharmacology (State-Province Key Laboratories of Biomedicine-Pharmaceutics of China, Key Laboratory of Cardiovascular Research, Ministry of Education,), College of Pharmacy, Harbin Medical University, Harbin, Heilongjiang 150081, P. R. China; 2 Northern Translational Medicine Research and Cooperation Center, Heilongjiang Academy of Medical Sciences, Harbin Medical University, Harbin, Heilongjiang 150081, P. R. China; 3 College of Bioinformatics Science and Technology, Harbin Medical University, Harbin, Heilongjiang 150081, P. R. China; 4 Department of Pharmacology and Therapeutics, Melbourne School of Biomedical Sciences, Faculty of Medicine, Dentistry and Health Sciences, University of Melbourne, Melbourne, Vic., Australia

## Abstract

Dysregulation of intracellular trafficking system plays a fundamental role in the progression of cardiovascular disease. Up-regulation of miR-1 contributes to arrhythmia, we sought to elucidate whether intracellular trafficking contributes to miR-1-driven arrhythmia. By performing microarray analyses of the transcriptome in the cardiomyocytes-specific over-expression of microRNA-1 (miR-1 Tg) mice and the WT mice, we found that these differentially expressed genes in miR-1 Tg mice were significantly enrichment with the trafficking-related biological processes, such as regulation of calcium ion transport. Also, the qRT-PCR and western blot results validated that Stx6, Braf, Ube3a, Mapk8ip3, Ap1s1, Ccz1 and Gja1, which are the trafficking-related genes, were significantly down-regulated in the miR-1 Tg mice. Moreover, we found that Stx6 was decreased in the heart of mice after myocardial infarction and in the hypoxic cardiomyocytes, and further confirmed that Stx6 is a target of miR-1. Meanwhile, knockdown of Stx6 in cardiomyocytes resulted in the impairments of PLM and L-type calcium channel, which leads to the increased resting ([Ca^2+^]_i_). On the contrary, overexpression of Stx6 attenuated the impairments of miR-1 or hypoxia on PLM and L-type calcium channel. Thus, our studies reveals that trafficking-related gene Stx6 may regulate intracellular calcium and is involved in the occurrence of cardiac arrhythmia, which provides new insights in that miR-1 participates in arrhythmia by regulating the trafficking-related genes and pathway.

Approximately 80% clinical cases of ischemic arrhythmia follow with sudden death^1^. The initiation and progression of arrhythmia depend upon electrical disorders in the heart^2^, but the mechanisms for electrical disorder remain unclear. Ion channels, including potassium, sodium and calcium, play essential roles in arrhythmia^3^,^4^. When suffering with ischemic stress, the cardiomyocytes (CMs) undergo lots of changes, especially the remodeling of ion channels of sarcolemma^3^,^5^. The mechanisms of ion channels organizing and targeting under pathophysiological conditions have been elucidated^6^. However, there is little information available about the role of intracellular trafficking in arrhythmia.

The intracellular trafficking was divided into three main types, which are forward (antegrade) trafficking to the membrane, channel behavior on the membrane, and reverse (retrograde) trafficking from the membrane^7^. In general, ion channels are synthesized, folded, and core-glycosylated in the endoplasmic reticulum (ER) and then subjected to quality control mechanisms^8^. When correctly folded, channels are transported to Golgi for the following processing, such as complex glycosylation^9^. Then channels are docked onto molecular motors and delivered to the plasma membrane by microtubule^10^. From the cell surface, channels become internalized and can either be recycled or degraded in proteasomes or lysosomes^11^. However, proteins themselves cannot transport or penetrate into cell membrane due to their structure and large size. The delivery of proteins depends on vesicles that can coat cargos and help them to transport. In addition, the vesicle transport is guided by specific recognition between vesicle membrane and its targets. Once there are some errors in the vesicle transport system or vesicle trafficking molecules, the intracellular homeostasis will be broken, even endanger the organ or body. It has reported that Tom70 (translocase of mitochondrial outer membrane 70) was down-regulated in pathological hypertrophic hearts, and acted as a molecular switch that orchestrates hypertrophic stress and mitochondrial responses to determine pathological cardiac hypertrophy^12^. Connexin 43 content and distribution were altered during experimental diabetes and likely contributed to alterations in cardiac function through oxidative modification of tyrosine mediated signaling^13^.

In recent years, microRNAs (miRNAs) has been reported to play a key role in physiology of muscles including heart^14^. miR-1 is considered to be muscle specific and is known to be involved in heart diseases such as hypertrophy, myocardial infarction, and arrhythmias^15^,^16^. In the previous study, our group found that miR-1 caused arrhythmia by post-transcriptional repressing ion channel-related gene and then disturbing the intracellular ion homeostasis for the first time^17^. In addition, we found that over-expression of miR-1 results in atrioventricular block in mice by causing intracellular Ca^2+^ imbalance^18^. However, its underlying mechanism is still unknown. This study was designed to examine whether intracellular trafficking contributes to the regulation of miR-1 on arrhythmia via regulating the transport of intracellular Ca^2+^.

## Results

### Distinct gene expression level emerges between miR-1 Tg mice and WT mice

**Figure 1:**
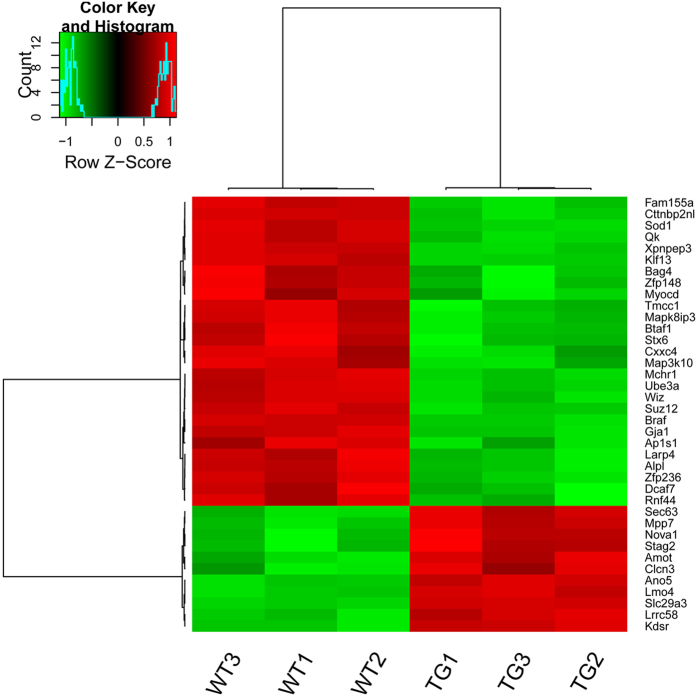
Heatmap of hierarchical clustering of differentially expressed targets of miR-1 in miR-1 Tg mice (TG) and wild type mice (WT). Red denotes high expression levels, whereas green depicts low expression levels.

Under the control of FDR < 0.05, 3417 genes were differentially expressed in the miR-1 Tg mice when compared with the WT group. Among the DEGs, 1438 genes were up-regulated and 1979 genes were down-regulated. In the gene expression profile, totally 787 genes were predicted to be targeted by the miR-1 in the TargetScan database. We found that 132 and 38 targets of miR-1 were significantly differentially expressed in the miR-1 Tg mice under the control of FDR < 0.05 and FDR < 0.01, respectively. Two distinctive expression clusters of those differentially expressed target genes were obtained by hierarchical clustering analysis. For clarity, we presented the heatmap derived by the 38 differentially expressed genes with FDR < 0.01 (Fig. 1).

### The targets of miR-1 are functionally enriched with ion-related process

**Figure 2:**
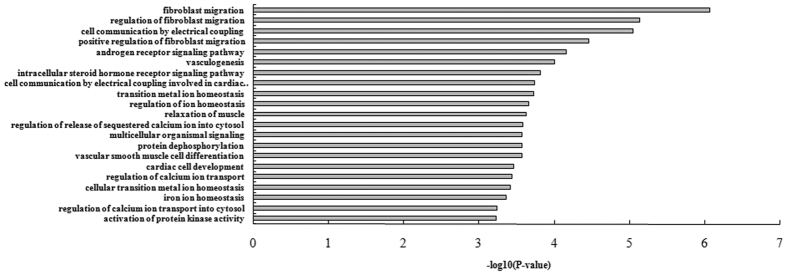
GO biological process terms with significant enrichment of differentially expressed miR-1 targets.

To investigate the function of the differentially expressed targets of miR-1 in the miR-1 Tg mice, we performed the biological process enrichment analysis using hygeometric model. Because the biological process terms from the Gene Ontology (GO) database with many annotated genes are too general to conduct specific biological meaning, we focused on analyzing the terms annotated with no more than 200 genes. Under the control of FDR < 0.05, we found the differentially expressed targets of miR-1 were significantly enriched in the 21 terms (Fig. 2). Using the GO analysis tool AmiGO (amigo.geneon-tology.org/cgi-bin/amigo/browse.cgi), the 21 BP terms were categorized into the following groups: ion homeostasis, cell communication, receptor signaling, protein kinase activation, vasculogenesis and fibroblast migration. As we known, ion channel is a main way of intracellular trafficking, and the process of ion channels synthesization, folding and transport to membrane are strictly controlled by intracellular trafficking system^19^. Scrutinizing the Fig. 2, the differentially expressed targets of miR-1 were mainly involved in the ion-related BP, including GO:0055076 (transition metal ion homeostasis, *P* = 1.86 × 10^−4^), GO:2000021 (regulation of ion homeostasis, *p* = 2.18 × 10^−4^), GO:0051279 (regulation of release of sequestered calcium ion into cytosol, *p* = 2.62 × 10^−4^), GO:0051924 (regulation of calcium ion transport, *p* = 3.60 × 10^−4^), GO:0055072 (iron ion homeostasis, *p* = 4.38 × 10^−4^) and GO:0010522 (regulation of calcium ion transport into cytosol, *p* = 4.38 × 10^−4^). Thus, we inferred that the differentially expressed targets of miR-1 in the miR-1 Tg mice behaved certain relationship with ion homeostasis in the trafficking system.

### The miR-1 regulates trafficking-related network

**Figure 3:**
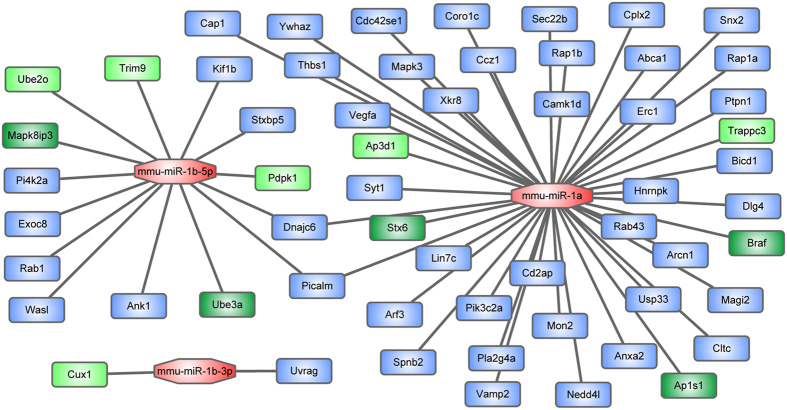
Network of miR-1 regulated trafficking-related genes. Round rectangle depicts miR-1 and octagon depicts targets of miR-1. The targets which were significantly down-regulated in the mice with over-expression of miR-1 (FDR < 0.05) were marked with green color and the targets with FDR < 0.01 were marked with dark green.

Then, vesicle-mediated transport (GO:0016192), a key pathway during the trafficking process, was significantly enriched with the differentially expressed genes in the miR-1 Tg mice (*p* = 4.48 × 10^−4^). Thus, we focused on analyzing the roles of miR-1 in regulating the pathway of vesicle-mediated transport. miR-1 regulated 58 genes in vesicle-mediated transport pathway (Fig. 3), and the result showed that targets of miR-1 were significantly enrichment with the vesicle-mediated transport pathway genes (*p* = 4.23 × 10^−3^). In Fig. 3, 11 of 58 the vesicle-mediated transport pathway genes were significantly down-regulated in miR-1 Tg mice (FDR < 0.05). The microarray results showed that Braf, Ube3a, Mapk8ip3, Stx6 and Ap1s1 were most significantly down-regulated in the miR-1 Tg mice (FDR < 0.01). Above results indicate that over-expression of miR-1 could inhibit the trafficking-related genes which participate in the vesicle-mediated transport pathway.

### Validation of differentially expressed target genes of miR-1 by quantitative RT-PCR and western blot

**Figure 4:**
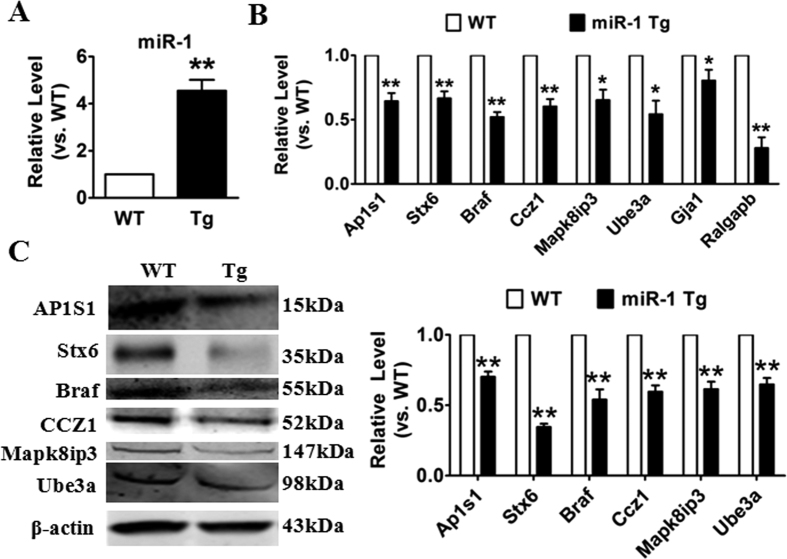
The dysregulation of trafficking-related genes in miR-1 Tg mice. (**A**) The expression of miR-1 in miR-1 Tg mice. qRT-PCR detection (**B**) and Western blot analysis (**C**) for trafficking-related genes in WT and miR-1 Tg mice. n = 6, **p < 0.01 *vs.*WT.

To further validate results derived from microarray, we performed qRT-PCR expression analysis for the most differentially expressed genes that were targeted by miR-1 in the vesicle-mediated transport pathway. We found that miR-1 was significantly up-regulated in miR-1 Tg mice (Fig. 4A). As shown in Fig. 4B, compared with the WT group, the mRNA levels of Ap1s1, Stx6, Braf, Ccz1, Mapk8i3p, Ube3a, Gja1 and Ralgapb were significantly decreased in miR-1 Tg mice, which indicated that miR-1 could blocked the trafficking-related genes. More importantly, the protein levels of these differentially expressed trafficking-related genes were dramatically lower in miR-1 Tg mice too (Fig. 4C). These data confirmed that miR-1 could regulate the trafficking-related genes.

### Acute ischemic or hypoxic insults reduced the expression of endogenous Stx6 in CMs

**Figure 5:**
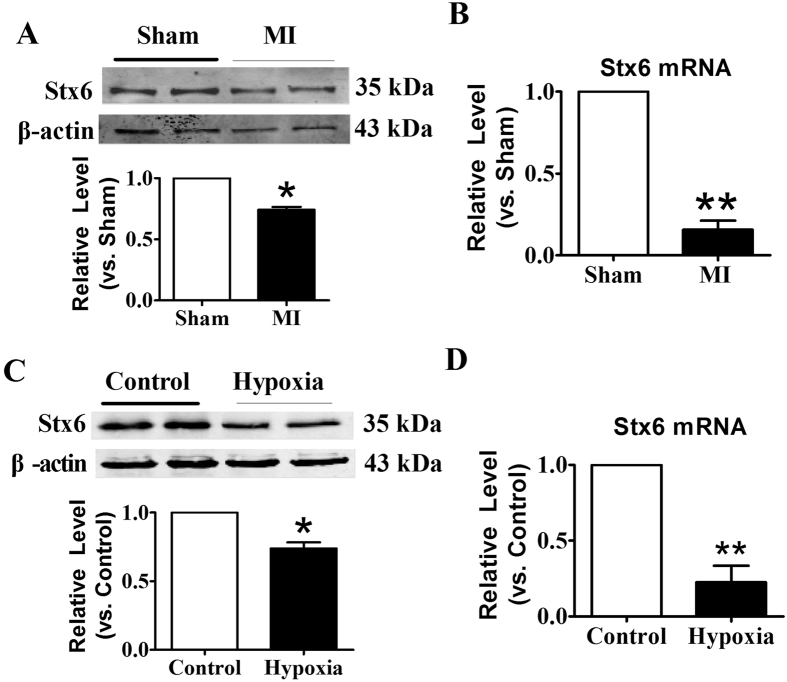
Stx6 was down-regulated in CMs subjected to ischemic or hypoxia stress. (**A**) Western blot detected Stx6 protein level in the hearts of mice under ischemic infarction for 12 hours. MI: myocardial infarction. (**B**) The mRNA expression level of Stx6 was down-regulated in the heart of mice under ischemic stress. Western blot (**C**) and qRT-PCR (**D**) were applied to detect the expression of Stx6 in the neonatal mice CMs which subjected to 12 hours of hypoxia with 1% O_2_. n = 6, *p < 0.05, **p < 0.01 *vs.* Sham or Control.

As a member of soluble N-ethylmaleimide-sensitive factor attachment protein receptor (SNARE) family, Stx6 has been found to be involved in the process of intracellular compartments^20^. We then investigated the role of Stx6 in ischemic cardiac arrhythmia model. As shown in Fig. 5A, the protein level of Stx6 was significantly decreased in the heart of mice subjected to MI compared with Sham group. Furthermore, the mRNA level of Stx6 was also markedly decreased in MI group (Fig. 5B). In accordance with the data from *in vivo*, the protein and mRNA levels of Stx6 were dramatically decreased in CMs which subjected to hypoxia for 12 hours (Fig. 5C & D). These results suggested that Stx6 played a role in ischemic cardiac arrhythmia, and which was sensitive to and down-regulated under ischemic stress.

### Stx6 is one of the targets of miR-1

**Figure 6:**
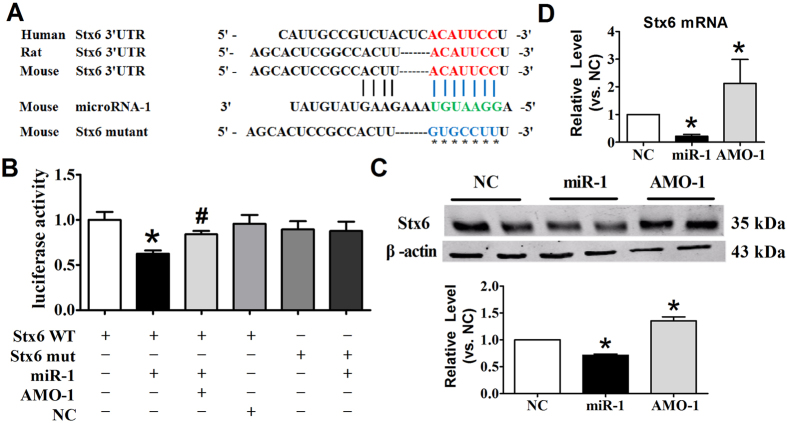
Stx6 is one of the direct targets of miR-1. (**A**) Conservation of the Stx6 3′UTR binding site for miR-1 among different species and the mutated binding sites of the Stx6 3′UTR. (**B**) Luciferase activity assay was used to measured the directly regulation of miR-1 on Stx6. Western blot (**C**) and qRT-PCR (**D**) were adopted to examine the effect of miR-1 on the protein and mRNA levels of Stx6. n = 6, *p < 0.05 *vs.*NC; #*p* < 0.05 *vs.*miR-1.

From the TargetScan database, we predicted the sequence position 73–79 in the 3′UTR of Stx6 as a putative miR-1 binding site (Fig. 6A). This sequence is broadly conserved in human, mouse and rat species. To identify whether Stx6 is a direct target of miR-1 through this specific binding site, we constructed a wild type luciferase reporter gene vector containing the 3′UTR of Stx6 or mutant vector with several mutations in the binding site. We then co-transfected the reporter with miR-1 mimics or miR-1 inhibitor (AMO-1) into HEK293 cells. Our data revealed that miR-1 could suppress the luciferase activity of wild-type Stx6, and this suppressive effect was reversed by the miR-1 inhibitor. However, the mutated form of Stx6 3′UTR demonstrated lesser response to miR-1 (Fig. 6B).

In addition, western blot and qRT-PCR analysis were applied to investigate the effects of miR-1 on Stx6 both at mRNA and protein level. Moreover, enforced expression of miR-1 led to a reduction of Stx6, and knockdown of endogenous miR-1 induced an increase in Stx6 expression both at protein and mRNA levels (Fig. 6C & D), indicating that miR-1 negatively regulated the Stx6 expression by transcriptional inhibition.

### Defect of Stx6 disturbs the intracellular Ca^2+^

**Figure 7:**
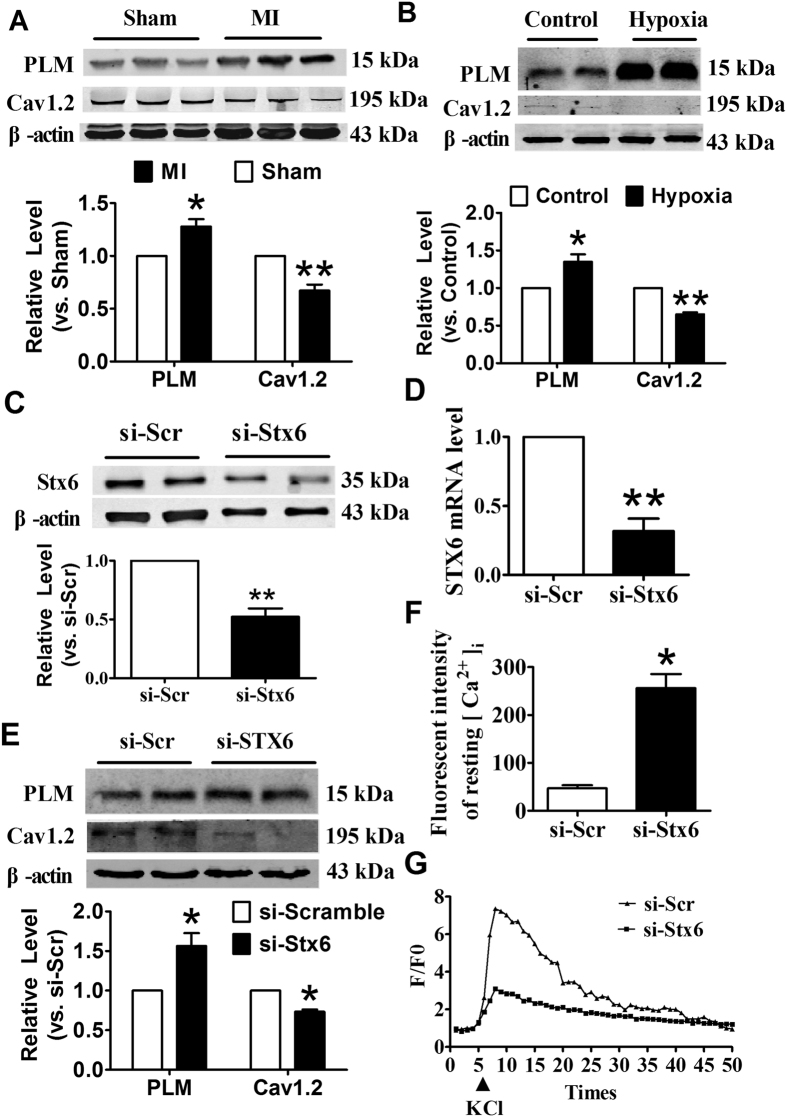
Stx6 participated in the regulation of intracellular Ca^2+^ in cultured CMs. (**A**) Western blot analysis for the protein levels of PLM and Cav1.2 in myocardial infarcted mice heart tissues. (**B**) The neonatal mice CMs were subjected to 12 hours of hypoxia with 1% O_2_, and western blot analyzed the protein level of PLM and Cav1.2. (**C**) Western blot analysis for Stx6 protein level at 48 hours after transfection with siRNA. (**D**) qRT-PCR detection for Stx6 mRNA level at 36 hours after transfection with siRNA. (**E**) Western blot analysis of the protein levels of Cav1.2 and PLM in Stx6-deficient neonatal mice CMs. (**F**) Resting [Ca^2+^]_i_ in empty plasmid and Stx6 knockdown neonatal mice CMs. (**G**) The linear graph showed confocal analysis for SR Ca^2+^ content changes induced by KCl (10 μM) in scramble and Stx6 knockdown neonatal mice CMs, F/F0 values represent Ca^2+^ transient. n = 6–9. *p < 0.05, **p < 0.01*vs.* MI or Control or si-Scr.

The imbalance of intracellular Ca^2+^ has been considered as an underlying mechanism of cardiac injury. And, increasing evidences showed that SNARE could interfere the homeostasis of intracellular Ca^2+^ 21. Then, we assumed that the defect of Stx6 contributes to ischemic heart disease by disturbing the intracellular Ca^2+^. To address this hypothesis, we firstly explored whether the expression of phospholemman (PLM), which directly interacts and suppress Na^+^/Ca^2+^ exchanger to regulate Cav1.2 and Ca^2+^ influx^22^,^23^, and Cav1.2 changed under cardiac injury. As shown in Fig. 7A, the PLM protein level was significantly increased under ischemic stress, whereas the expression of Cav1.2 was dramatically decreased in ischemic heart tissues. The CMs were subjected to oxygen deficit, which resulted in PLM increasing and Cav1.2 decreasing (Fig. 7B). These data confirmed that ischemia and hypoxia could break the balance of intracellular Ca^2+^, and induce cardiac injury.

To examine the potential regulatory effect of Stx6 on intracellular Ca^2+^ homeostasis, we generated a siRNA construct for Stx6 that was able to inhibit expression of Stx6 both at protein and mRNA expression levels in CMs (Fig. 7C & D). Then, we tested the PLM and Cav1.2 protein levels when the Stx6 was inhibited. As shown in Fig. 7E, compared with si-scramble group, knockdown of Stx6 markedly increased the protein level of PLM in CMs. On the contrary, inhibition of Stx6 resulted in decreased expression of Cav1.2 (Fig. 7E). Furthermore, we performed confocal analysis to evaluate the intracellular Ca^2+^ change. Compared with si-scramble group, silence of Stx6 significantly increased the resting ([Ca^2+^]_i_) in CMs (Fig. 7F). More importantly, the sensitivity to KCl stimulation was lower in the group with knockdown of Stx6 than that in si-scramble group (Fig. 7G). However, the time to peak of F/F0 between two groups showed no significant differences. Taken together, these data suggested that knockdown of Stx6 would damage intracellular calcium system of CMs.

**Figure 8:**
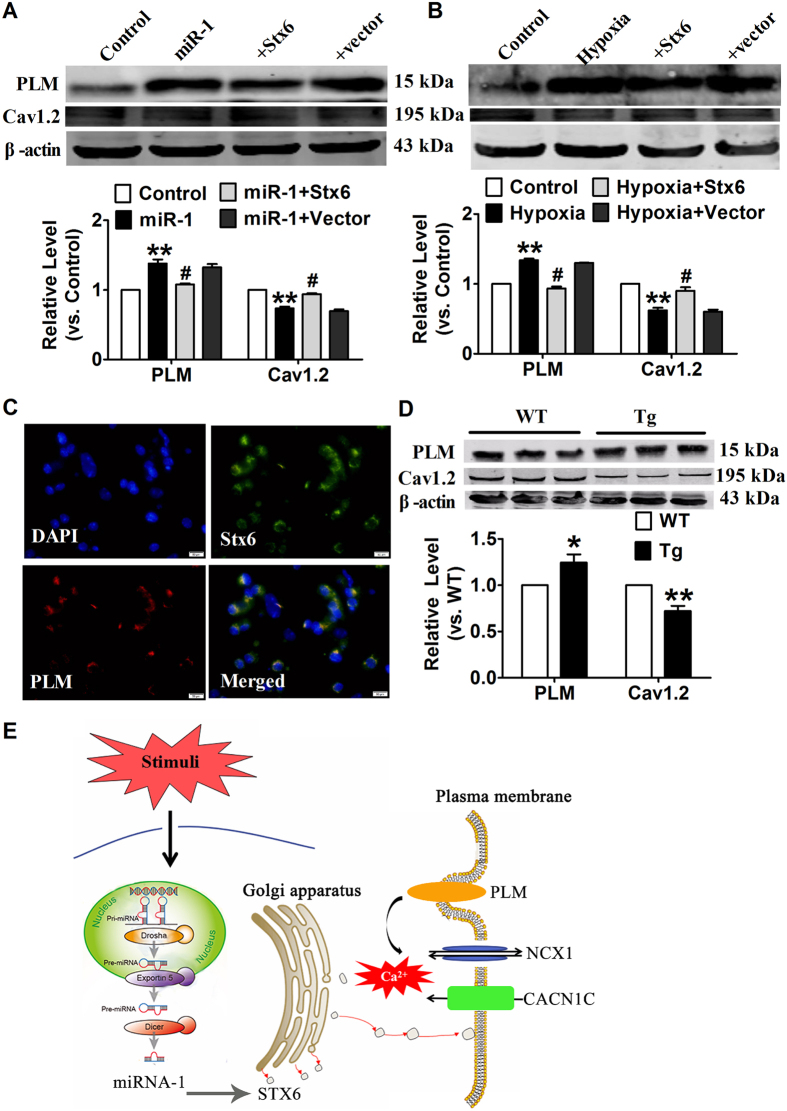
Over-expression of miR-1 broke the balance of Ca^2+^, and induced cardiac arrhythmia. Neonatal mice CMs were transfected with Stx6 during overexpression of miR-1 (**A**) or hypoxia (**B**), and western blot was applied to analyze the protein levels of PLM and Cav1.2. (**C**) Immunofluorescent localization of Stx6 and PLM in neonatal mice CMs. Green represent staining of Stx6, red represent staining of PLM, blue represent staining of nuclei. (**D**) Western blot analysis for the protein levels of PLM and Cav1.2 in WT and miR-1 Tg mice. (**E**) Schematic depiction of the results and proposed hypothesis. n = 4−6. **p < 0.01 *vs.*Control, WT or NC; #*p* < 0.05 *vs.*miR-1 or Hypoxia.

We then transfected Stx6 into CMs to further explore the role of Stx6 in cardiac calcium. As shown in Fig. 8A and B, overexpression of miR-1 or response to hypoxia in CMs resulted in the up-regulation of PLM and down-regulation of Cav1.2, whereas forced expression of Stx6 alleviated these effects of miR-1 or hypoxia. These results indicated that Stx6 improve intracellular calcium system of CMs, and which may be a novel strategy for the treatment of arrhythmia.

Finally, our data showed that there is a co-localization between Stx6 and PLM in the cultured CMs, indicating that Stx6 regulates Ca^2+^ homeostasis by interacting with PLM (Fig. 8C). Meanwhile, we investigated the effect of miR-1 on expression of PLM and Cav1.2. As expected, miR-1 Tg mice exhibited an increase of PLM protein expression level and decrease of Cav1.2 protein level (Fig. 8D). These data indicated that over-expression of miR-1 resulted in the imbalance of intracellular Ca^2+^ by modulating PLM through direct regulation of Stx6, and then contributed to arrhythmia in mice.

## Discussion

In this study, we investigated the role of trafficking-related molecules in miR-1-driven cardiac arrhythmia in mice model. Firstly, we found that the differentially expressed genes in miR-1 Tg mice were significantly enrichment with the trafficking-related biological processes, and up-regulation of miR-1 significantly inhibited trafficking-related genes. Second, our study suggested that the logistics molecules Stx6 is a direct target of miR-1 and participated in the process of miR-1-induced arrhythmia. Finally, our results revealed that the broken down of Stx6 under ischemic stress improved the expression of PLM while decreased Cav1.2 expression, and then led to the overload of intracellular Ca^2+^, and finally triggered arrhythmia (Fig. 8E).

More and more efforts are performed to reveal the underlying molecular mechanism of arrhythmia and develop novel therapeutic strategy for arrhythmia. It has reported that intracellular trafficking plays key roles in lots of physiological and pathological processes including cardiac fatty acid utilization^24^, inflammatory bowel diseases^25^ and prion diseases^26^. Increasing evidences have showed that vesicle trafficking molecules make a contribution to cardiovascular diseases^27^,^28^. SNX13 reduction can activate pro-apoptotic caspase and promote apoptosis repressor degradation, which results in heart failure^27^. Dynamin-2 deficiency caused CMs apoptosis through promoting SR calcium overload^28^. Thus, we suspected whether the changes of trafficking systems contributed to the pathogenesis of arrhythmia.

MiR-1 is an important regulator of heart adaption after ischemic stress and is up-regulated in patients with myocardial infarction^17^,^29^. In the previous study, we found that miR-1 is a pro-arrhythmia factor by regulating GJA1 (gap junction alpha-1 protein), KCNJ2 and intracellular calcium^17^,^18^,^30^. However, the exact role of miR-1 in regulating the trafficking process is still unclear. Thus, we considered whether miR-1 participates in the intracellular trafficking process by regulating trafficking-related genes. We performed bioinformatic and molecular biological methods to explore the role of miR-1 in intracellular trafficking process. We found that some trafficking-related genes were deregulated in miR-1 Tg mice, indicating that vesicle-mediated transport pathway may contribute to the process of miR-1-induced arrhythmia in mice. In addition, the critical role of lncRNAs in the process of trafficking has been reported. Thus, investigation of lncRNAs in regulating trafficking-related pathways deserves our future detailed study.

SNARE proteins on the vesicle membranes plays key roles in intracellular materials transport. And, Stx6 is a member of SNARE protein family, which was found to participate in lots of intracellular compartments. So far, Stx6 has been found to interact with VAMP2, VAMP4, VAMP7 and VAMP8, and contributes to exocytosis^31^. Stx6 is mainly reported to mediate a trans-Golgi network (TGN) trafficking towards endosome^32^. However, there is a low but significant level of Stx6 on 3T3L-1 cell membrane^33^. Thus, we inferred that Stx6, as a member of trafficking molecules, takes part in multiple fusion events. In this study, we found that Stx6 was decreased in MI tissues and hypoxic CMs and negatively regulated by miR-1. These studies indicated that Stx6 might serve as a potential target for ischemic arrhythmia.

Increasing evidences support that SNARE proteins together with L-type calcium channel participate in exocytosis and endocytosis through regulation of Ca^2+^ fluxes in intramembrane system^34^,^35^. Then, the question raised here is whether the intracellular Ca^2+^ contributes to the effect of Stx6 in arrhythmia.

PLM, a member of FXYD family, is a regulator of the Cav1.2 and Ca^2+^ influx^36^. In rat myocytes, PLM over-expression alters contractility and cytoslic [Ca^2+^]_i_ transients^37^. PLM mimics depresses the activity of NCX1, which causes Ca^2+^ overload and CMs death^38^. In this study, we found that knockdown of Stx6 could activate the expression of PLM and depress Cav1.2 expression. Furthermore, Stx6 deficiency increased the resting [Ca^2+^]_i_, and sensitivity to KCl stimulation was lower in Stx6 knockdown group, which showed Stx6 defect resulted in Ca^2+^ overload and CMs damage. Furthermore, we performed MI and CMs hypoxia model to verify the PLM and Cav1.2 changes during cardiac injury. We demonstrated that cardiac injury indeed could increase PLM level and decrease Cav1.2 level, indicating the effect of Stx6 on ischemic arrhythmias. In addition, we found that over-expression miR-1 could increase the PLM level and decrease the Cav1.2 level both *in vivo* and *in vitro*. These results indicated that miR-1 caused the imbalance of Ca^2+^ homeostasis in CMs at least partly via down-regulation of Stx6. It has reported that the stimulation of β-adrenergic receptor (β-AR) could activate protein kinase A (PKA) to enhance cardiac inotropy and lusitropy^39^. PLM, as a substrate of PKA, can be activated and regulated by PKA^23^. And, our results present the co-localization between Stx6 and PLM. Thus, there are two hypotheses for how Stx6 regulates PLM expression: (1) Stx6 directly decreases the expression of PLM; (2) Stx6 changes the activity of PKA and indirectly regulates the expression of PLM. More studies should be done to verify these hypotheses.

## Materials and Methods

### Ethics statement

The study was approved by the Institutional Animal Care and Use Committee of Harbin Medical University, P.R. China (No. HMUIRB-2008–06). All experimental procedures were performed in accordance with the Guide for the Care and Use of Laboratory Animals, published by the US National Institutes of Health (NIH Publication No. 85–23, revised 1996).

### Microarray analysis

The generation of cardiomyocytes-specific over-expression of microRNA-1 (miR-1 Tg) mice was described in previous study^18^. Total RNA was extracted from three miR-1 Tg mice and three wild type (WT) mice, respectively. Microarray detection was performed using Affymetrix GeneChip Mouse Gene 1.0 ST array. The probe-level data in CEL files were converted into gene level expression. We performed background correction and quartile data normalization by the robust multiarray average (RMA) algorithm with defaulted parameters in R affy package. When multiple probes were mapped to the same gene ID, the mean value was used to represent the expression value of the single gene. We used empirical Bayes method, which is ComBat function from Bioconductor, to remove the batch effect that may have occurred during processing of the arrays^40^.

### Differentially expressed genes (DEGs) analysis

The two-tailed T-test was used to identify genes that were significantly differentially expressed between miR-1 Tg mice and WT mice. The *P* values were adjusted by the Benjamini and Hochberg (BH) correction procedure to account for multiple tests. And, the genes with the false discovery rate (FDR) < 1% were defined as differentially expressed genes (DEGs).

### Identification of predicted miRNA targets and Gene Ontology (GO) enrichment analysis

The predicted target mRNAs of miR-1 were downloaded from the TargetScan database with release version 6.2 (http://www.targetscan.org/cgi-bin/targetscan/data_download.cgi?db=mmu_61). Hierarchical clustering of expression values of differentially expressed miR-1 targets was performed with R software using the metric of Euclidean distance and average linkage. The GO database contains three categories: biological process, molecular function and cellular component. We used the hypogeometric distribution model to test whether the differentially expressed targets of miR-1 were significantly enrichment with the biological processes (BP). The BH procedure was used to adjust the *P* values. FDR < 0.05 were used as a threshold to select significant GO terms. The trafficking-related network was drawn by cytoscape (http://www.cytoscape.org/).

### Quantitative real-time PCR (qRT-PCR)

Total RNA was isolated from cultured cardiomyocytes using a trizol standard protocol (Invitrogen, Carlsbad, USA). The integrity, quantity, and purity of RNA were examined using Nano-Drop 8000 Spectrophotometer (Thermo Scientific, Wilmington, USA). For each sample, 500 ng of total RNA was converted to cDNA using High Capacity cDNA Reverse Transcripition Kit (Applied Biosystems, Foster City, USA). The relative expression levels of mRNAs and miRNAs were quantified by qRT-PCR with SYBR Green I (Applied Biosystems). After circle reaction, the threshold cycle (Ct) was determined and relative mRNA and miRNA levels were calculated based on the Ct values and normalized to GAPDH or U6 level in each sample.

### Western Blot

For western blot analysis, total protein samples were extracted from tissues or cells with the procedure as previously described^41^. Briefly, tissues or cells were lysed with RIPA lysis buffer with complete protease inhibitor cocktail (Roche Molecular Biochemicals, Basel, Switzerland). 100 μg proteins were fractionated on a 8% or 15% SDS-PAGE. After electrophoretically transferring to a Pure Nitrocellulose Blotting membrane (Pall Life Science), the blots were probed with primary antibodies, with β-actin (Proteintech, Rosemont, IL, USA) as an internal control. Primary antibodies against PLM, Stx6 and Cav1.2 were purchased from Proteintech (Rosemont, IL, USA). The immunoreactivity was detected using Odyssey Infrared Imaging System (Gene Company Limited, Hongkong, China). The bands were quantified by measuring the band intensity for each group.

### Myocardial Infarction model

Male C57BL/6 mice, 20–30 g, were randomly divided into sham and myocardial infarction (MI) groups. MI was established as described^42^. The mice were anesthetized with 2,2,2-tribromoethanol and placed in the supine position with the upper limbs taped to the table. A 1~1.5-cm incision was made along the left side of the sternum. The muscle layers of the chest wall were bluntly dissected to avoid bleeding. The thorax was cut open at the point of the most pronounced cardiac pulsation and the right side of the chest was pressed to push the heart out of the thoracic cavity. The left anterior descending coronary artery was occluded and then the chest was closed. All surgical procedures were performed under sterile conditions. Twelve hours after occlusion, the cardiac tissues within the ischemic zone, border zone and non-ischemic zone distal to the ischemic zone were dissected for the following experiments. Sham mice underwent open chest procedures without coronary artery occlusion.

### Cell culture

Neonatal mice CMs were prepared as previously described^43^. CMs were cultured in Dulbecco’s modified Eagle’s medium (DMEM; Hyclone, Logan, UT, USA) containing 10% fetal bovine serum (FBS; Hyclone). CMs were purified by differential plating and added 0.1 mmol/L BrdU (5-bromo-2-deoxyuridine) was to eliminate the cardiac fibroblasts. Cells were cultured in a 5% CO_2_ and 37 °C humidified atmosphere, and CMs were seeded in six-well plates and cultured for forty-eight hours. CMs were transfected with 100 nmol/L miR-1, with X-treme GENE siRNA transfection reagent. Forty-eight hours after transfection, cells were used for RNA or protein purification. Induction of hypoxia, CMs were placed in a hypoxic chamber which was kept at 37 °C for twelve hours, and a constant stream of water-saturated 94% N_2_, 5% CO_2_, and 1% O_2_ were maintained.

### Luciferase assay

HEK293 cells (American Type Culture Collection, ATCC, Manassas, VA) were cultured with DMEM containing 10% FBS and 1% penicillin/streptomycin. HEK293 cells were co-transfected in 24-well plates with the Stx6 WT or mutant constructs and miR-1, using the Lipofectamine 2000 reagent (Invitrogen, Carlsbad, CA, USA). After 48 h of transfection, the cells were harvested and lysed and the luciferase activity was assayaed using the Dual Luciferase Reporter Assay System (Promega) according to the manufacturer’s instructions. The firefly luciferase values were normalized to renilla, and the relative ratios of firefly to renilla activity were reported.

### Confocal imaging

CMs were stained by 2 μM Fluo-3/AM at 37 °C for 1 h. And then, the cells were washed with standard external solution containing calcium for three times. Fluorescent images were captured by laser scanning confocal microscope (Olympus America Inc., Center Valley, USA) with 488 nm for excitation and 530 nm for emission. Free intracellular Ca^2+^ level was presented as F_0_, and sarcoplasmic reticulum (SR) Ca^2+^ load was measured by applying KCl (10 μM).

### Immunofluorescent cell staining

CMs in 24-well plates were washed briefly with cold PBS for 3 times and fixed with 4% paraformaldehyde for 15 min. Then, the cell membrane was penetrated by Triton X-100 for 1 h and blocked by normal goat serum for 1 h at 37 °C. The cells were incubated with anti-Stx6 antibody (Abcam) and anti-PLM (Proteintech, Rosemont, IL, USA) overnight at 4 °C. Next day, the cells subsequently incubated with FITC-conjugated goat anti-mouse or goat anti-rabbit antibody for 1.5 h. The cells were washed with PBS, stained nuclei with DAPI (Roche Molecular Biochemicals) for 5 min at room temperature. Immunofluorescence was analyzed under a fluorescence microscope.

### Statistical analysis

Bioinformatic analyses were performed using R software, version 3.0.0 (http://www.R-project.org). All the data are expressed as the mean ± SEM. GraphPad Prism 5.0 was used for the statistical analysis. For two treatment groups, the Student *t* test was used. For three or more treatment groups, one-way ANOVA was used with Bonferroni post-test for the comparison of selected two treatment groups as well as Dunnett post-test for comparing all other treatment groups to the corresponding control. A value *p* < 0.05 was considered statistically significant.

## Additional Information

**How to cite this article:** Su, X. *et al*. Over-expression of microRNA-1 causes arrhythmia by disturbing intracellular trafficking system. *Sci. Rep.* **7**, 46259; doi: 10.1038/srep46259 (2017).

**Publisher's note:** Springer Nature remains neutral with regard to jurisdictional claims in published maps and institutional affiliations.
